# Diabetic Nephropathy and Gaseous Modulators

**DOI:** 10.3390/antiox12051088

**Published:** 2023-05-12

**Authors:** Subir Kumar Juin, Rosemary Ouseph, Dibson Dibe Gondim, Venkatakrishna Rao Jala, Utpal Sen

**Affiliations:** 1 Department of Physiology, University of Louisville School of Medicine, Louisville, KY 40202, USA; 2 Department of Microbiology & Immunology, Brown Cancer Center, Center for Microbiomics, Inflammation and Pathogenicity, University of Louisville School of Medicine, Louisville, KY 40202, USA; 3 Division of Nephrology & Hypertension, University of Louisville School of Medicine, Louisville, KY 40202, USA; 4 Department of Pathology, University of Louisville School of Medicine, Louisville, KY 40202, USA

**Keywords:** diabetic nephropathy, nitric oxide, carbon monoxide, hydrogen sulfide

## Abstract

Diabetic nephropathy (DN) remains the leading cause of vascular morbidity and mortality in diabetes patients. Despite the progress in understanding the diabetic disease process and advanced management of nephropathy, a number of patients still progress to end-stage renal disease (ESRD). The underlying mechanism still needs to be clarified. Gaseous signaling molecules, so-called gasotransmitters, such as nitric oxide (NO), carbon monoxide (CO), and hydrogen sulfide (H_2_S), have been shown to play an essential role in the development, progression, and ramification of DN depending on their availability and physiological actions. Although the studies on gasotransmitter regulations of DN are still emerging, the evidence revealed an aberrant level of gasotransmitters in patients with diabetes. In studies, different gasotransmitter donors have been implicated in ameliorating diabetic renal dysfunction. In this perspective, we summarized an overview of the recent advances in the physiological relevance of the gaseous molecules and their multifaceted interaction with other potential factors, such as extracellular matrix (ECM), in the severity modulation of DN. Moreover, the perspective of the present review highlights the possible therapeutic interventions of gasotransmitters in ameliorating this dreaded disease.

## 1. Introduction

### 1.1. Diabetic Nephropathy

Diabetic nephropathy (DN) is one of the leading causes of end-stage renal disease (ESRD) in developed and developing countries and is predicted to grow to 20–30% of the patients with type 1 diabetes (T1D) and type 2 diabetes (T2D) combined. The various risk factors responsible for the development of renal disease in individuals with renal dysfunction include the time span of diabetes, age at diagnosis, race, poor glycemic control, hypertension, genetic susceptibility, and dietary composition, among others [1,2,3,4]. However, the precise pathogenic mechanisms associated with the initiation and progression of DN remained incompletely understood. One of the hallmarks of DN is the progressive expansion of the mesangial matrix, which is developed by the accumulation of the components of the extracellular matrix (ECM) [5]. Alteration in local gene expression of humoral growth factors, such as transforming growth factor-β (TGF-β), connective tissue growth factor (CTGF), and platelet-derived growth factor (PDGF), may promote elevated production of the ECM component, e.g., fibronectin and collagen IV, or decreased degradation by matrix metalloproteinases, e.g., MMP-1 and MMP-2, in DN [6,7,8].

DN in humans undergoes several distinct pathophysiological changes, including an early stage of glomerular hyperfiltration, which is followed by the so-called silent phase when the glomerular filtration rate (GFR) becomes normal [9]. Subsequent development of microalbuminuria, dipstick-positive proteinuria, and thereafter a continuous decrease in the GFR leads to ESRD [10,11].

**Figure 1:**
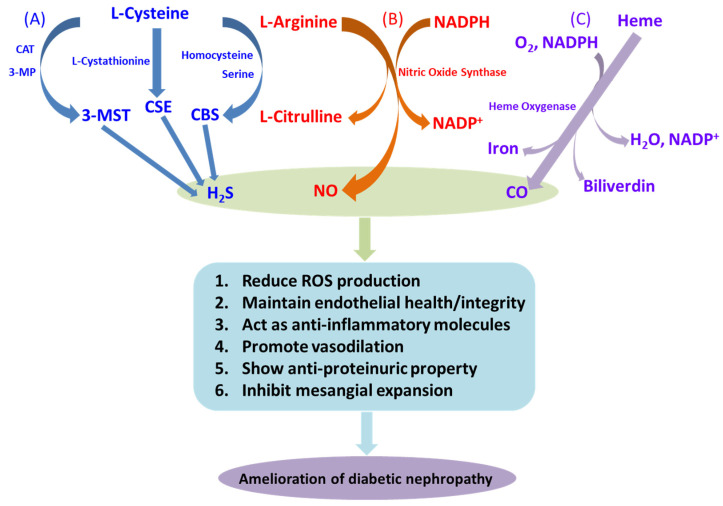
Diabetic nephropathy and gaseous molecules. Schematic representations of the pathway of synthesis of the gasotransmitters and their beneficial effects in diabetic nephropathy: (**A**) H_2_S is synthesized from L-cysteine by the enzymatic action of cystathionine β-synthase (CBS) and cystathionine γ-lyase (CSE), as well as by the combined action of 3-mercaptopyruvate sulfurtransferase (3-MST) and cysteine aminotransferase (CAT). (**B**) NO is synthesized by the catalytic activity of nitric oxide synthase (NOS) via a series of redox reactions, with degradation of L-arginine to L-citrulline in the presence of NADPH. (**C**) In the presence of functional heme oxygenase (HO), the porphyrin ring of heme is broken and oxidized to produce CO, ferrous iron, and biliverdin. These gasotransmitters exert several responses, some of them mentioned in the figure, which help to prevent deleterious effects of DN.

Nonetheless, the pathogenesis of DN is a multifactorial disease where hyperglycemia initiates and triggers a number of pathophysiological events. Recent advances in diabetes research provide us with many key insights into DN at the molecular and cellular level that involve oxidant and antioxidant balance, extracellular matrix turnover, matrix metalloproteinases and their tissue inhibitors, gap junction proteins, noncoding RNAs, and the microbiome, to name a few. In addition, a variety of gasotransmitters, such as CO, NO, and H_2_S, play a vital role in the development and progression of DN (Figure 1). In light of the current literature, we summarize the biology of these gaseous molecules and their interaction and involvement in modulating DN in this review. In the end, we also discuss their potential therapeutic implications to intervene this devastating disease.

### 1.2. Gaseous Molecules (CO, NO, and H_2_S) and DN

#### 1.2.1. Carbon Monoxide (CO) and DN

Over the years, carbon monoxide (CO) has emerged as a gasotransmitter that is produced by the different heme oxygenases (HOs) as a product of heme metabolism [12]. There are three different isoforms of HO, viz., the inducible form, HO-1, and the two constitutive isoforms, HO-2 and HO-3. Among the three isoforms, HO-1 and HO-2 are physiologically active, while the physiological relevance of HO-3 is yet to be confirmed [13,14]. In the kidney, HO-1 and HO-2 render cytoprotection and act as physiologic regulators of heme-dependent protein synthesis. HO converts heme into biliverdin, iron, and CO. Various physiological functions have been assigned to CO, such as vasodilation and inhibition of platelet aggregation. In skeletal muscle and leukocytes from T2D patients, HO-1 mRNA was found to be dramatically decreased compared to that of non-diabetic controls [15,16]. Contrarily, in spite of an upregulated HO-1 expression, a reduction in the vasorelaxant function of CO was observed in STZ-induced T1D rats [17]. CO production was found to be decreased in aortic tissue in Zucker diabetic fatty (ZDF) rats, compared to that of controls. Moreover, increasing HO-1 activity with cobalt protoporphyrin resulted in elevated CO, which contributed to the decreased glucose levels and enhanced insulin sensitivity in ZDF rats [18]. These findings suggest that increased insulin sensitivity might mediate reduced vascular risk in the presence of elevated CO levels [18]. Hemin, an inducer of the HO pathway, was found to be protective against renal inflammation and facilitated the amelioration of DN [19,20,21]. The antioxidant effect of HO-1 seems to render renoprotection in diabetes [22]. On the contrary, HO-2 deficiency leads to increased superoxide anion and renal dysfunction following STZ-induced diabetes [23]. Thus, induction of HO-1 and -2 activity has been beneficial to improve glucose metabolism and mitigate DN by attenuating hyperglycemia-induced oxidative injury [22,23].

In a nutshell, a reduced CO level is accompanied by insulin resistance and a reduction in endothelial health, whereas an elevated level of CO remains beneficial in DN [24]. These findings clearly suggest a plausible role of the HO-1/CO pathway, which can be exploited for therapeutic intervention to restrict the development and progression of diabetes and its complications. The effects of CO in DN are graphically represented in Figure 1C.

#### 1.2.2. Nitric Oxide (NO) and DN

Nitric oxide (NO) is a short-lived lipophilic gaseous molecule produced in almost all tissues and organs and involved in different biological functions under physiological and pathological conditions. NO is a paracrine regulator, which was initially recognized as an endothelium-derived relaxing factor [25]. It is endogenously produced from its substrate L-arginine by three distinct nitric oxide synthase (NOS) enzymes, i.e., neuronal, inducible, and endothelial NOSs (nNOS or NOS-1, iNOS or NOS-2, and eNOS or NOS-3, respectively) [26]. All three forms of NOS are expressed by the kidney [27]. The nNOS resides in neurons and skeletal muscle cells, and it mediates important neuronal cell–cell interactions [28]. The iNOS remains in the vascular system and is predominantly active in the immune system under oxidative stress and promotes inflammation [29]. In the kidney, iNOS is produced in the proximal tubules and medulla during inflammation or sepsis and may lead to oxidant injury [30]. The eNOS is expressed in the arterioles and glomerular capillaries and is mainly involved in maintaining and regulating vascular tone [27,31]. NO has been recognized to function as a vasodilator, inhibits platelet aggregation, and stabilizes atherosclerotic plaques [32].

In diabetes, endothelial dysfunction leads to the impaired production of vascular NO [33], and endothelial NO synthase gene (eNOS) polymorphisms have been identified in a meta-analysis [34]. In addition, an association between eNOS polymorphisms leading to reduced eNOS expression and the development of advanced nephropathy in T1D [35,36] and T2D patients has been reported [37]. Contrarily, other studies did not find any potential link between eNOS polymorphisms and DN [38,39,40].

However, dysfunctional eNOS has been shown to act as a common pathogenic pathway in diabetic vascular complications, although the functional mechanism is unclear. In induced diabetic eNOS KO mice, a study showed that hyperglycemia severity was similar to diabetic WT mice. In contrast, the diabetic eNOS KO mice developed overt albuminuria, hypertension, and glomerular mesangiolysis compared to diabetic WT and non-diabetic control mice [41]. In addition, a significant reduction in glomerular hyperfiltration, endothelial injury thickened GBM, and effacement of the focal foot process in the diabetic eNOS KO mice were also observed [41]. These findings indicate a pivotal role of NO in the pathogenesis of DN.

Additionally, differential production of NO has been evidenced in DN. Although an increase in intra-renal NO synthesis was observed in the early stages of DN, a progressive decline in renal production, as well as the bioavailability of NO, was reported in the advanced stages of renal failure [42]. In the serum of DN patients with microalbuminuria, significantly higher concentrations of NO end products, i.e., nitrite/nitrate, have been reported [43]. Increased NO level either indicates an upregulated inflammatory response by iNOS or a protective response against eNOS-mediated renal injury. Deficiency of eNOS leading to accelerated nephropathy in diabetic mice [44,45] also supports a protective role for NO in DN [46]. Moreover, in T2D rats, supplementation of a NOS cofactor, tetrahydrobiopterin (BH4), mitigated renal damage [47]. Reduced eNOS expression and NO production have been suggested as the rationale for impaired NO-dependent vasodilatation in T2D patients [48,49]. In a rat model, blockade of NOS results in insulin resistance, indicating that loss of NO synthesis precedes T2D [50]. Reduced NO production was observed in spontaneous as well as streptozotocin (STZ)-induced T1D rats [51,52]. The therapeutic effect of a NO donor, molsidomine, was demonstrated in STZ-induced DN in rats [53]. In the T2D mouse model, NO’s bioavailability is reduced, resulting in endothelial dysfunction and impairment in the NO-mediated vasodilatation [54,55]. Apart from these protective effects, NO is an important regulator in inducing nitrosative stress and inflammation in diabetes. Therefore, NO plays a dual role in the development and progression of diabetes and vascular dysfunction [56]. Some modes of NO action in DN are depicted in Figure 1B.

The above findings clearly reveal that NO production is differentially modulated in DN, and the lower expression of this gasotransmitter indicates a significant regulatory role in DN. Enhancement of the redox potential by scavenging the ROS may be indicated as the mechanistic insight of these findings. NO-based interventions have already been applied in humans. Sodium nitroprusside (SNP) is clinically used as a direct NO donor without any need for enzymatic action [57]. Nitroglycerin and other organic nitrates are also well-established for their vasodilatory effects [58]. Organic nitrates act as NO donors by breaking down nitrates into nitrite and NO [58]. Molsidomine and linsidomine have been registered in many European countries as vasodilators by the non-enzymatic release of NO. Moreover, high nitrate-rich dietary products can act as NO donors to reduce blood pressure. For example, the intake of beetroot juice significantly lowers blood pressure, accompanied by higher levels of total urinary nitrite/nitrate [59].

#### 1.2.3. Hydrogen Sulfide (H_2_S) and DN

Over the last three decades, hydrogen sulfide (H_2_S) has overcome its past reputation as a toxic gas and gained much attention as a molecule of various biological roles spanning from neurotransmission, vasorelaxation [60], nociception [61,62], cytoprotection [63,64], cardiovascular modulation [65], atherosclerosis [66], and ischemia-reperfusion injuries [67] to diabetes complications [68,69]. In mammalian tissue, H_2_S is synthesized from L-cysteine by two cytosolic pyridoxal 5′-phosphate (PLP)-dependent enzymes, i.e., cystathionine β-synthase (CBS) and cystathionine γ-lyase (CSE) [70,71,72]. A PLP-independent enzyme 3-mercaptopyruvate sulfurtransferase (3MST) has also recently been identified to produce H_2_S from 3-mercaptopyruvate [73,74] (Figure 1A).

A significant amount of H_2_S is produced in various mammalian tissues. H_2_S concentrations in the brain of mammals, including cows, rats, and humans, were found to be very high, as high as 46 µM in serum and 50–160 μM in the brain of rats [75], though later it was suggested that these recorded concentrations were seemingly high due to the lack of standardized measurement methods [76]. Despite the controversy over the actual H_2_S concentration present in blood, it is generally accepted that H_2_S acts as an endogenous regulator of vasorelaxation and cardiovascular function [77,78]. H_2_S is also regarded as the first gaseous KATP channel opener, since H_2_S injection triggered a transient yet significant reduction in mean arterial blood pressure, which was antagonized by the application of a specific K_ATP_ channel blocker, i.e., glibenclamide, and mimicked by pinacidil, a specific K_ATP_ channel opener [79,80]. Thus, the hypotensive effect of H_2_S was supposed to be stimulated by the relaxation of resistance blood vessels through the opening of K_ATP_ channels. Although the mechanism of K_ATP_ channel opening is not clear, it does not influence the concentration of ATP. KATP channel activity is mainly involved in insulin secretion. KATP channel opening of the pancreatic β cells inhibits insulin secretion, whereas its closure augments the secretion. Though H_2_S acts as the gaseous KATP channel opener, it has no channel specificity, and therefore, H_2_S exhibits some effect on insulin secretion.

In humans, diabetes is associated with lower levels of H_2_S. In a group of patients having T2D, median plasma H_2_S levels were found to be decreased by 73% compared to those in healthy individuals [81]. It is noteworthy to mention that obesity is correlated with lower levels of H_2_S compared to those of healthy individuals. Taken together, human and experimental diabetes are associated with decreased H_2_S bioavailability, which might be linked to increased cardiovascular risk, as observed in diabetic patients.

Contrarily, elevated H_2_S concentration in Zucker diabetic rats indicates that H_2_S remains high during insulin resistance conditions [82]. Similarly, streptozotocin-induced diabetic rats showed elevated production of H_2_S in the pancreas [83] and increased expression of H_2_S-producing enzymes [84,85]. Although the nonspecific K_ATP_ channel opening activity of H_2_S evokes some inhibitory effect on insulin secretion, the H_2_S level remains high in hyperinsulinemia. On the other hand, we found that H_2_S-producing enzymes are markedly lowered in the kidney of Ins2^Akita^ diabetic mice [86]. Others have found similar results in T1D patients [87]. Moreover, in spontaneously hypertensive rats, intraperitoneal injection of exogenous H_2_S reduces blood pressure and prevents the progression of DN [88]. In STZ-induced T1D mice, intraperitoneal H_2_S administration attenuated oxidative stress, inflammation, and mesangial cell proliferation [89]. However, these seemingly conflicting results need to be confirmed by further evidence, and there remains a consensus that H_2_S is associated with diabetic disease conditions.

Unfortunately, H_2_S has not yet been clinically used in humans, albeit intravenous Na_2_S being administered in a phase 1 trial [90]. However, thiosulfate is used for the treatment of end-stage renal disease [91], and it shows a protective role in a mice model of heart failure [92] and hypertensive heart and renal disease in rats through H_2_S generation [93,94]. In addition, zofenopril and captopril, the sulfhydrylated ACE inhibitors, showed additional beneficial responses in the trials [95], and the beneficial effects of sulfhydrylated ACE inhibitors have been recently explained by the H_2_S release [96]. Since sulfate-reducing bacteria produce H_2_S in the gut and significantly lower levels of H_2_S were observed in germ-free mice [97], the dietary supplementation of sulfate or sulfur-containing amino acids may act as natural H_2_S donors. Thus, H_2_S may be an excellent tool to treat various disease conditions depending on the relative abundance of H_2_S availability associated with the specific disease states, for example, DN.

In Table 1, we summarized the experimental models, their intervention strategies, whether their levels were increased or decreased, overall outcomes, and the cited references of all three gasotransmitters.

#### 1.2.4. DN and Polysulfides

Exogenous as well as endogenously derived H_2_S is stored in the tissue as bound sulfane sulfur through sulfuration [100,101,102]. Endogenously bound sulfane sulfur was observed in several tissues, including the brain and liver [101,102], but to date, it has not been explored whether bound sulfane sulfur is protein specific and under which physiological condition release of bound H_2_S is regulated. Recently, the role of garlic-derived polysulfide production and its prospective physiological relevance in cardiovascular protection through H_2_S and NO was elucidated [103]. It has been presumed that garlic-derived polysulfides, viz., diallyl sulfide, diallyl disulfide, and diallyl trisulfide, are the potent H_2_S donors, which facilitate increased bioavailability of NO through phosphorylation of eNOS, leading to cardiovascular protection [104]. However, a similar renoprotective role of garlic-derived polysulfides and simultaneous intervention of H_2_S in DN may be a subject of interest in future research.

## 2. Receptor-Mediated DN and Gaseous Molecules

### 2.1. NMDA Receptors, Diabetes, and Gaseous Molecules (CO, NO, and H_2_S)

The *N*-methyl-D-aspartate receptor (NMDA-R) is a heterotetrameric protein complex that functions as a membrane calcium channel. In mammals, functional NMDA-Rs consist of an obligatory subunit NMDA-R1 (NR1) interacting with a second class of subunits of proteins NR2A-NR2D, which provide the functional variability of the receptor [105,106,107,108]. The existence of renal NMDA-Rs has been confirmed through immunoblot, immunostaining, and renal hemodynamic studies in rat kidney cortexes [109]. It has also been demonstrated that significant functional inhibition of the renal NMDA-R is not connected to the central nervous system effects [109]. Instead, renal NMDA receptors have been reported to stimulate proximal reabsorption and glomerular filtration, and inhibition of these receptors resulted in distinct renal vasoconstriction and reduction in renal blood flow [110].

A confirmatory role for renal NMDA-R in maintaining normal renal function has also been reported, suggesting that the activation of NMDA-R mediates the renal response to glycine infusion. The requirement of the NMDA-R for the co-agonist glycine gives reliability to the latter suggestion, as does the ability of systemically administered NMDA-R inhibitors to selectively alter renal hemodynamics [111]. Moreover, inhibitors of the NMDA-R attenuated successive glycine response independent of their effects on the baseline renal blood flow. Renal sympathetic nerve activity may lead to renal vasoconstriction [112], while centrally active NMDA antagonists may augment peripheral sympathetic activity [109].

Available information on the NMDA-R antagonists indicates that these drugs do not cross the blood–brain barrier, and no evidence for generalized sympathetic overactivity in these experiments was obtained [109,113]. In addition, renal denervation did not modify the renal response to either NMDA-R inhibitor. Immunostaining revealed that NMDA-R remains in proximal tubules, where they are positioned to account for the effects of NMDA-R antagonists on basal renal blood flow and the GFR by increasing the tubular reabsorption and decreasing the macula densa signal for the tubuloglomerular feedback. The micropuncture study revealed that increased tubular reabsorption accounts for nearly half of the vasodilatory response to the glycine infusion [114,115]. However, the existence of NMDA-R in other renal cells may be discovered in future research.

NO primarily mediates glutamate action at NMDA-Rs, while CO is mainly involved in glutamate effects at metabotropic receptors [12]. However, studies have revealed that CO may be involved in the glutamate and NMDA-agonist-induced vasodilation of newborn pig cerebral arterioles. The study further suggested that CO-induced cerebral vasodilation can be dependent on NO action [116]. However, to our knowledge, the role of CO in the modulation of NMDA receptors in diabetes has not been studied.

The activation of NMDA leads to calcium entry and stimulates the activity of neuronal NO synthase (nNOS). The major agonists, glutamate and glycine, facilitate the activation and opening of the channel. In addition, renal blood flow/GFR response to the common agonist, glycine, which generally enhances renal blood flow, was abrogated in the rats pretreated with different NMDA-R antagonists [117]. These findings indicate that glycine-induced activation of the NMDA-R in the kidney may lead to vasodilation via NO effects or indirectly by modifying agonist activity, such as angiotensin II [118]. It is worth mentioning that although nNOS is expressed in the kidney and influences glomerular hemodynamics, future research may confirm whether the hemodynamic effects of the renal NMDA-R are intervened via nNOS. Moreover, linkage to nNOS in arcuate/interlobular arteries appears to be unexpected. Detailed studies are necessary to unravel the downstream consequences of the NMDA-R in the future [109].

**Figure 2:**
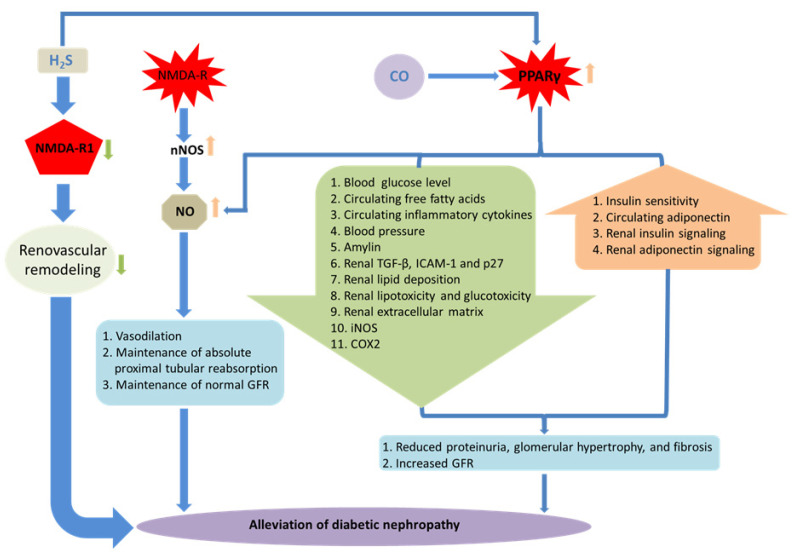
Receptor-mediated DN and gaseous molecules. Schematic representations of the role of gaseous molecules in receptor-mediated DN. Elevated expression of NMDAR-1 induces pathophysiological changes leading to the DN, while H_2_S treatment ameliorates such effects. Activation of NMDA-R stimulates neuronal NO synthase (nNOS) leading to the synthesis of NO, which mitigates pathophysiological changes in diabetic kidney and maintains normal renal functions. H_2_S and CO can activate PPARγ, which helps in the alleviation of renovascular remodeling and confers renal protection. Together, renoprotection is also associated with the activation of PPARγ, simultaneous increase in NO production, and reduction in systemic blood pressure.

NMDA receptors are one of the major targets of H_2_S in the brain. It has been reported that H_2_S specifically potentiates the activity of NMDA-Rs and facilitates the induction of hippocampal long-term potentiation (LTP), which appears to have a protective role in cognitive decline during aging and neurodegenerative disorders [70]. The basal level of NMDA-Rs maintains normal kidney function, while elevated expression may induce pathophysiological changes [119]. It has been observed that NMDA-Rs are stimulated in acute kidney injury [120]. The NMDA-R1 subunit is the main subunit responsible for the channel activity of NMDA-Rs, which is predominant in renal glomeruli and proximal tubules [121]. Along the same line, we reported that elevated expression of NMDA-R1 in both mRNA and protein levels was observed in the diabetic kidney as well as in high glucose-induced mouse glomerular endothelial cells (MGECs) [86]. We also reported that higher expression of NMDA-R1 was associated with a lower level of H_2_S in diabetic conditions [86]. Furthermore, through an in vitro study, we demonstrated that supplementation of H_2_S mitigated NMDA-R1 expression in HG [86]. More recently, we have also shown that NMDA-R1 mediates Ca^2+^ influx, which results in the activation of cyclophilin D and opening of the mitochondrial permeability transition pore leading to the oxidative outburst and renal endothelial injury, while H_2_S treatment mitigates NMDA-R1 expression and thus prevents renal damage [122]. Therefore, it is plausible that H_2_S may mitigate NMDA-R1 expression and ameliorate diabetic renal remodeling. A possible link of NMDA-R, NO, CO, and H_2_S in DN is depicted in Figure 2.

### 2.2. PPARγ, Diabetes, and Gaseous Molecules (CO, NO, and H_2_S)

The peroxisome proliferator-activated receptor-γ (PPARγ) is a member of the steroid/thyroid nuclear receptor superfamily of ligand-activated transcription factors. PPARγ is predominantly expressed in adipose tissues and plays a critical role in adipocyte differentiation, fat deposition, and glucose and lipid homeostasis [123,124]. Expression of PPARγ at low levels has been observed in many non-adipose tissues along with the vasculature and kidney [125,126], suggesting that PPARγ might be playing a crucial role in renal function and regulation of blood pressure.

Over the last decade, growing evidence has suggested that activation of PPARγ is involved with the attenuation of DN. Apart from their effects on the amelioration of insulin resistance and T2D, synthetic ligands of PPARγ, i.e., TZDs (thiazolidinediones), have emerged as a promising drug to reduce proteinuria and mitigate the progression of DN, irrespective of glycemic control [127,128,129]. TZDs also mediate direct anti-atherogenic effects in the diabetic vasculature independent of their metabolic actions [130]. In the pathogenesis of diabetic vasculopathy, such as glomerulosclerosis, downregulated PPARγ expression is associated with matrix accumulation and glomerulonephritis [124]. Numerous studies have elucidated the efficacy of PPARγ agonists in ameliorating the progression of glomerulosclerosis [131] and have indicated the direct involvement of PPARγ ligands in renoprotection [132].

Previous studies reported the intimate functional relationships between PPARγ and gaseous molecules, such as NO and CO [133,134]. Renoprotective effects of PPARγ were found to be associated with the modulation of the release of vasodilator substances, such as NO [135,136]. PPARγ activation has also been demonstrated in response to CO [134]. The activation of HO/CO/PPARγ signaling was shown to play a critical role in the manifestation of the beneficial effect of PPARγ agonist pioglitazone against the cyclosporine-induced detrimental effect on renovascular activity [136]. This study also highlighted the therapeutic potential of CO or NO donors in the management of cyclosporine A (CsA)-induced impaired renal vasodilation [136].

In a relatively recent study, we reported that ciglitazone, a PPAR agonist, was found to ameliorate DN by reducing glomerular tissue homocysteine (Hcy), which is also a precursor of H_2_S [137]. We also reported that H_2_S could prevent hyperhomocysteinemia (HHcy)-induced renal failure by regulating MMP-2, -9, and collagen in mice [138,139]. Our recent study revealed that H_2_S supplementation by GYY4137 reinstated decreased PPARγ levels and improved adverse ECM remodeling in type 1 DN [140]. Therefore, a therapeutic intervention involving gasotransmitters may pave the way for the treatment of DN by regulating PPARγ in the future. A possible link of PPARγ, NO, CO, and H_2_S in DN is depicted in Figure 2.

## 3. Matrix Remodeling in DN: Role of Gaseous Molecules

### 3.1. Structural Protein (Collagen and Elastin) Regulation by Gaseous Molecules in DN

During the development and progression of DN, glycation of the extracellular matrix (ECM) leads to the deposition of the ECM proteins in the mesangium, renal tubulointerstitium, and glomerular basement membranes (GBMs) [141]. Increased expression of ECM causes thickening of the GBM as well as the tubular basement membrane (TBM) and expanded mesangial matrix, leading to glomerulosclerosis and tubulointerstitial fibrosis [141]. Therefore, the accumulation of ECM proteins plays an important role in the development of DN.

**Figure 3:**
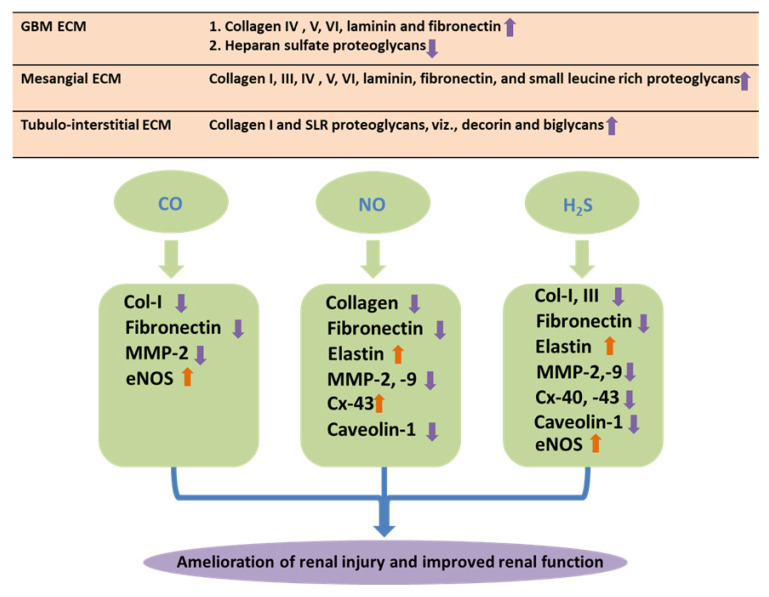
Matrix protein and gaseous molecules. Schematic representations of role of matrix proteins and their differential regulations by gaseous molecules in DN. During development of DN, deposition of the ECM proteins in the mesangium, renal tubulointerstitium of the glomerulus, and the glomerular basement membranes (GBMs) leads to renal fibrosis. Gasotransmitters, i.e., CO, NO, and H_2_S, facilitate amelioration of the adverse effect of matrix remodeling through differential regulations of the matrix proteins during DN.

The ECM glycoproteins that are increased in DN include collagen, laminin, fibronectin, and proteoglycans. Initially, glycation affects the interactions of collagen with the cells and other matrix components, but the most damaging effects are caused by the formation of glucose-mediated intermolecular cross-links, which greatly hampers the critical flexibility and permeability of the tissues and reduces turnover. The principal perturbations of ECM components in the GBM include upregulation of collagen IV (α3 and α4 chains), V, VI, laminin, and fibronectin, while there is a downregulation in heparan sulfate proteoglycans [141,142,143]. In addition, the changes in the ECM proteins of the tubulointerstitial compartment include elevated expression of collagen I and small leucine-rich (SLR) proteoglycans, viz., decorin and biglycans [144]. On the other hand, mesangial matrix changes comprise increased expression of collagen I, III, IV (α1 and α2 chains), V, VI, laminin, fibronectin, and SLR proteoglycans [141,142]. As collagen and elastin are the two major structural protein components in the ECM, the changes in these proteins and the role of gaseous molecules in DN are discussed below. In addition, the modulation of several other ECM proteins by their gaseous regulators NO, CO, and H_2_S are depicted in Figure 3.

#### 3.1.1. Collagen and Gaseous Molecules in DN

The collagen family of proteins is the most abundant in humans and provides the framework for the most vulnerable tissues in the kidney, such as the renal basement membrane. The optimal functioning of the kidney tissues is dependent on the integrity of their supporting framework of collagen.

An earlier study demonstrated that the exogenous application of a low dose (250 ppm) of CO in a glass exposure chamber inhibits the development of renal fibrosis in obstructive nephropathy by attenuating the induction of key ECM proteins, such as type 1 collagen, in mice [145]. Moreover, it has also been demonstrated that the low dose of CO treatment inhibits progressive chronic allograft nephropathy by reducing collagen 1 in rats [146].

Previous reports showed that arginine increases plasma levels of nitrate/nitrite in diabetic patients [147]. Arginine has been shown to increase nitrates and exhale NO in both control as well as insulin-dependent diabetes mellitus (IDDM) patients [148]. Moreover, it was also indicated that l-arginine inhibits collagen accumulation in the kidney [149], heart [150], and GBM [151] of diabetic mice and also in advanced-stage glycosylation end products (AGEs) [151,152]. As the AGEs are reported to quench NO [153], arginine supplementation may appear to be beneficial to improve endothelium-dependent vasodilation by inhibiting AGE-mediated mitigation of NO-dependent relaxation [147].

A previous study revealed that H_2_S donors, such as sodium hydrosulfide (NaHS), inhibit the renal fibrosis of obstructive nephropathy by attenuating the accumulation of collagen fibrils in the renal interstitium in rats [154]. Supplementation with H_2_S has been shown to mitigate renal damage in hypertensive models by reducing blood pressure, proteinuria, and oxidative stress and inhibiting excessive collagen type I and collagen type III deposition [93,155,156]. In a murine model, H_2_S supplementation has also been reported to prevent HHcy-induced glomerulosclerosis by regulating collagen [139]. Recently, H_2_S has been demonstrated to ameliorate renal tissue fibrosis and the development of DN by inhibiting excessive collagen deposition in STZ-induced diabetic rats [157].

#### 3.1.2. Elastin and Gaseous Molecules in DN

Elastin is a 70 kDa glycoprotein, and it constitutes the central core of elastic fibers. Elastin provides support and elasticity, which are important for many tissues and organs, such as the blood vessels, heart, skin, lungs, and uterus. The cross-linked and random-coiled structure of elastin renders the capacity of the elastic network to stretch and recoil. A unique glycoprotein microfibril, Fibrillin, has been recently identified to be associated with elastic fibers in compliant tissues [158]. Elastin is not considered to be a primary component of the capillary BM. Notably, the capillary tuft of the glomerulus is devoid of elastin, and it is present only in the mesangial stalk as well as in afferent and efferent arterioles [159]. This may be one of the reasons why the capillary BM of the glomerular tuft undergoes remodeling expansion and causes thickening of its BM when exposed to intra-glomerular hypertension, which occurs early in the natural history of T2D.

It has been reported that NO donors, such as S-nitrosoglutathione, facilitate a multi-fold increase in the synthesis and deposition of ECM protein elastin in a dose-dependent manner [160]. Another study exhibited that NO delivery dose-dependently stimulates tropoelastin synthesis to increase vascular elasticity [161]. These studies indicate that NO supplementation may ameliorate the adverse effect of renovascular remodeling during DN.

A recent study demonstrated that H_2_S might attenuate vascular calcification by upregulating elastin levels through inhibition of the Stat3/CAS signaling cascade during hyperglycemia [162]. Homocysteine (Hcy), which induces elastinolytic proteinase in VSMCs [163], has been reported to cause arterial stiffness by modulating the elastin/collagen ratio, resulting in hypertension [164] and diabetes [165]. Moreover, HHcy has been shown to decrease H_2_S [166] and increase MMPs, which induce the degradation of elastin [167,168,169]. In the hypertensive and diabetic mouse models, HHcy-induced activation of MMPs was shown to be normalized by oral or intraperitoneal H_2_S supplementation, leading to the prevention of renal damage [137,138,140,170]. Therefore, H_2_S treatment could be a promising therapeutic approach to prevent renovascular damage by attenuating the MMP-mediated degradation of elastin.

### 3.2. Proteinases and Their Inhibitors’ (MMPs and TIMPs) Regulation by Gaseous Molecules in DN

Matrix metalloproteinases (MMPs) are a family of zinc-dependent endopeptidases that are involved in the breakdown and remodeling of ECM components [171]. The abnormal activity of these endopeptidases is associated with a variety of vascular diseases, including cardio-pulmonary and renovascular [172,173]. Research findings suggest that hyperglycemia abnormally affects the expression and activity of MMPs in diabetic kidneys [174].

Currently, 28 different types of MMPs have been discovered in vertebrates [175]. Of these, at least 23 mammalian MMPs have been recognized, and these MMPs were further subdivided into different groups [176,177]. Structural analysis revealed that MMPs are multi-domain proteins that generally consist of a prodomain, a catalytic domain, a hinge region, and a hemopexin domain in the case of collagenases, gelatinases, and membrane-type MMPs (MT-MMPs). MMPs are generally secreted as nonfunctional pro-MMPs, which are activated either by cleavage of the prodomain by different proteinases, such as plasmin and MT-MMPs, or by oxidation of reactive cysteine within the prodomain [178,179]. MT-MMPs are a typical class of MMPs with a broad spectrum of activities and remain anchored to the cell surface by the transmembrane domains. MT-MMPs are believed to predominantly regulate proteolytic activities within the pericellular microenvironment due to their presence on the cell surface [180].

The expression of several MMPs and tissue inhibitors of metalloproteinases (TIMPs) in the nephron of various species has already been discussed elsewhere [141,181]. Sub-cellular localization of protein expression of several MMPs, including MMP-2, -3, -9, -10, -11, -14 (MT1-MMP), -15 (MT2-MMP), TIMP-2, and TIMP-4, have been reported and summarized in human kidney tissues [182,183]. As the MMPs play a major role in the glomerular ECM degradation and turnover, the alteration in expression and activity of the MMPs influences the intra-renal extracellular matrix composition [184,185]. Renal hypertrophy, which is developed early in T1D, predominantly occurs in individuals who develop DN later and is implicated in poor renal prognosis [186,187,188]. As the unusual ECM accumulation is one of the hallmarks of DN, it is plausible that changes in MMP expression and activation may contribute to DN, especially to the advent of renal hypertrophy. It is noteworthy that, apart from the direct role in ECM turnover, MMPs secrete or activate numerous growth factors, viz., tumor necrosis factor-α, pro-transforming growth factor-β, insulin-like growth factors, and heparin-binding-epidermal growth factor, which are involved in renal hypertrophy, tubular cell proliferation, renal scarring, and kidney fibrosis [189,190,191,192].

The role of MMPs in DN is critical in the earlier phases of the disease progression when increased matrix accumulation, the release of pro-fibrotic growth factors, and altered cell motility disrupt the glomerular and tubular architectures. Therefore, an in-depth understanding of the role of MMPs in the pathogenesis of DN is essential for the therapeutic intervention of MMPs in preventing and mitigating diabetic kidney disease. Below, we discuss the involvement of MMP-2 and -9 and their regulation by CO, NO, and H_2_S in DN.

#### 3.2.1. Gelatinases (MMP-2 and MMP-9)

In numerous studies, it has been shown that dysregulation in intra-renal gelatinase plays an important role in kidney diseases. For example, it has been demonstrated that intra-renal MMP-2 expression is increased in AL-amyloidosis [193] and human renal carcinoma [194]. It has also been shown that MMP-2 is essential for instigating the transformation of renal tubular cell epithelium–mesenchymal transformation, which is a critical step in forwarding the progression of renal interstitial fibrosis in several kidney diseases, including DN [195,196]. In reality, over-expression of MMP-2 in renal proximal tubular epithelial cells was demonstrated to develop the characteristic pathologic changes of chronic kidney disease [197].

A contrasting relationship between MMP-2 dysregulation and DN was revealed. The decreased expression and/or proteolytic activity of MMP-2 and increased activity of the MMP-2 inhibitor, TIMP-2, were observed in renal tissues of the rodent diabetic models [198,199,200]. On the other hand, both the increase as well as a decrease in MMP-2 production or activity have been observed in rodent mesangial cells cultured under hypoglycemic conditions [201]. However, in human studies, an increase in MMP-2 association and activity was evidenced in DN [202,203]. In addition, the upregulation of MT5-MMP, which contributes to the activation of MMP-2, was observed in diabetic kidney tissue in humans [204]. An elevation in urinary MMP-2 concentrations and/or MMP-2 activity was shown in albuminuric patients having T1D compared to that of controls as well as non-albuminuric patients [205,206].

In a murine model of T2D, MMP-9 expression in the kidneys of mice that developed nephropathy was increased compared to controls [207]. In addition, an elevated level of MMP-9 has been observed in the urine of patients with T2D and DN, and the level of MMP-9 was found to be increased in congruence with the extent of albuminuria [208,209]. Injury or apoptosis in the podocyte has been identified as a part of renal disease processes characterized by the failure of the filtration barrier [210,211]. Cultured podocytes have been shown to produce MMP-2 and MMP-9, which can be influenced by various cytokines, growth factors, and hyperglycemic conditions [212]. Recently, hyperglycemia-induced apoptosis and depletion of podocytes have been demonstrated in murine T1D and T2D models [213].

Interestingly, podocytopenia occurs early in diabetic patients with T1D and T2D [214,215,216]. A hypothetical reduction in podocyte density could be achieved by glomerular basement membrane expansion, secondary to MMP-induced alterations of the ECM turnover. While coupled with hyperglycemia-induced podocyte injury and increased apoptosis of the podocytes, a distinct increment in membrane permeability would result, leading to diabetic albuminuria. The appearance of podocytes in the urinary sediment of diabetic patients having albuminuria compared to the absence of podocytes in the normoalbuminuric T1D patients corroborates this hypothesis [217]. These patients’ plasma MMP-9 levels were significantly correlated with the number of urinary podocytes. Therefore, these findings indicate that diabetes-associated gelatinase dysregulation may perturb podocyte integrity and permeability of the glomerular basement membrane [217].

It is noteworthy to mention that the CO-releasing molecule CORM-2 was found to inhibit MMP-2 activities in the alveolar epithelial cells [218]. NO has been shown to modulate the cytokine (IL-1β)-induced expression of MMP-9 and also regulate the enzymatic activity of MMP-9 in a rat mesangial cell culture [219]. It has also been demonstrated that NO regulates MMP-9 expression in rat mesangial cells through a post-transcriptional mechanism [220]. NO-mediated post-transcriptional regulation of MMP-9 was also evidenced in rat mesangial cells [221]. In addition, an increased NO level has been shown to modulate MMP-2 and -9 activation in the diabetic feto-placental unit [222].

Our own studies revealed that supplementation of H_2_S prevents HHcy-associated renal damage by regulating MMP-2 and MMP-9 in mice [138,139]. An in vitro study also demonstrated that H_2_S supplementation marginally attenuated but could not completely normalize MMP-9 levels in hyperglycemic conditions [86]. Recently, our group showed that an H_2_S donor, GYY4137, could ameliorate ECM accumulation and renal fibrosis by downregulating MMP-9 expression either via miR-194-mediated inhibition of ROS production or through modulation of PPARγ and retinoid X receptor signaling in type 1 DN [99,140].

#### 3.2.2. Tissue Inhibitors of Metalloproteinases (TIMPs)

Decreased serum levels of TIMP-1 and TIMP-2 have been observed in patients with T2D and DN compared to diabetes alone or non-diabetes chronic renal failure [223]. On the contrary, in the younger T1D patients with normal kidney function, TIMP-1 or TIMP-2 concentrations remained unchanged compared to that of age-matched non-diabetic controls [205]. Differences in the disease severity and duration or differences in the pathophysiology of T1D and T2D may explain these contrasting clinical observations.

Elevated plasma levels of HO-1 and TIMP-4 have been demonstrated as potential markers of pathogenesis in T2DM with tuberculosis [224]. It has been demonstrated that NO regulates TIMP-1 in rat mesangial cells [219]. Moreover, H_2_S supplementation by GYY4137 has been shown to regulate TIMP-1 expression in mouse kidney mesangial and glomerular endothelial cells [225]. A recent study showed that H_2_S intervention alleviates renal fibrosis and may play a protective role against the development of DN by regulating TIMP-1 in STZ-induced diabetic rats [157].

### 3.3. Gap Junction Regulation by Gaseous Molecules in DN

Gap junctions are formed by the members of the connexins (Cxs) protein family [226]. The association between two Cxs in the plasma membrane of adjoining cells gives rise to a functional gap junction channel facilitating cell-to-cell communication [227]. Among twenty distinct types of connexins, human and mouse kidneys have been reported to express eight isoforms of Cxs, viz., Cx26, 30, 32, 37, 40, 43, 45, and 46 [228]. A recent study revealed that H_2_S ameliorates the expression of Cx40, Cx43, and Cx45 in diabetic animal models [86,228]. Differential regulations of connexins, i.e., upregulation of Cx40 and downregulation of Cx37 and Cx43, have been reported to act in conjunction with eNOS to modulate vascular function in diabetes [229,230]. Le Gal and colleagues showed a distinct role of the CX40-mediated regulation of NO production in a hypertensive mouse model [231]. On the other hand, the role of NO in the regulation of gap-junction-mediated intercellular communication has also been reported in the mesangium. Yao et al. (2005) demonstrated that elevated NO augments CX43-mediated gap junctional intracellular communication in mesangial cells via protein kinase A and that decreased NO may cause loss of CX43-mediated cell communication in the mesangium in DN [232].

### 3.4. Other Integral Membrane Proteins’ (Caveolin and eNOS) Regulation by Gaseous Molecules

Caveolin is crucial for the formation of caveolae membranes, which act as scaffolding domains. The caveolin family consists of three caveolins, i.e., caveolin-1, -2, and -3 [233,234]. Caveolin-1 and -2 co-express as well as form a hetero-oligomeric complex in the many cell types [235,236], while caveolin-3 is muscle-specific [237]. Generally, caveolin-1 and -3 have higher regulatory activity than caveolin-2.

Caveolin-1 can induce caveolae formation, while caveolin-2 cannot induce the formation of caveolae. Therefore, generally, caveolin-1 is considered the principal structural protein of caveolae [238]. In recent years, the presence of caveolae and caveolin-1 and their roles in the kidney have been demonstrated in several studies [238,239,240,241,242,243]. Thus, controlling the proliferation ability of the mesangial cells could be an effective therapy for kidney diseases [243]. In fact, exogenous CO administration, as well as adenoviral-mediated HO-1 expression, enhanced the association between caveolin-1 and toll-like receptor-4 (TLR4), leading to the generation of anti-inflammatory response [244]. These findings identify the HO-1-mediated interaction between caveolin-1 and TLR4 as the potential therapeutic targets for inflammatory diseases [245].

In a mouse model of DN in T1D, caveolin-1 deficiency has been reported to render protection against mesangial matrix expansion [246]. Caveolin-1 expression was found to be upregulated in the glomeruli of patients with glomerular disease, including DN [238]. Elevated caveolin-1 plays a critical role in the suppression of eNOS-mediated renal NO production, which is presumably responsible for the progression of DN [98]. However, treatment with a NO donor, such as sodium nitrite, or NO precursor, such as L-arginine, ameliorated the adverse effects of DN [98].

In endothelial cells, caveolin-1 is the principal structural component of caveolae. Caveolin-1 acts as a scaffolding protein and is involved in the modulation of receptor signaling and the function of the caveolar enzymes [247,248]. eNOS is inhibited by its protein–protein interaction with caveolin-1 in the unstimulated endothelial cells. The course of eNOS activation by the stimulation of an agonist involves intracellular Ca^2+^ mobilization and a subsequent interaction between calmodulin and eNOS. The eNOS/calmodulin interaction allows the release of eNOS from the inhibitory complex with caveolin-1 [249,250]. Thus, changes in caveolin-1 abundance and eNOS interactions may influence eNOS activity and, subsequently, vascular function and modeling. A comprehensive knowledge and understanding of the role of caveolin-1 in mediating the cellular functions in diabetes are requisite for the interpretation of NO pathophysiology in the diabetic kidney. In diabetes, although ROS-mediated inactivation of NO has been suggested as the key mechanism behind the decreased bioavailability of eNOS-derived NO [251,252], other relevant mechanisms involved in the direct changes in eNOS function and molecular integrity have also been proposed [253]. Moreover, the expression of renal cortical eNOS regarding some of its functional determinants, such as cellular localization, phosphorylation status, and dimer/monomer formation, has been explored in normal and diabetic rats [254]. Furthermore, renal cortical expressions, as well as localization of the endogenous eNOS inhibitor caveolin-1 and its colocalization with eNOS, have been revealed.

During HHcy-induced renal injury, exogenous supplementation of H_2_S dehomocysteinylated eNOS and reduced caveolin-1 to increase eNOS availability, resulting in the inhibition of renovascular fibrosis and improved renal function [170]. However, to our knowledge, the role of H_2_S in DN has not yet been reported in the literature. Future studies might shed light on whether H_2_S regulates caveolin and ameliorates kidney injury and function in diabetes.

## 4. Summary and Future Perspectives

The gasotransmitters CO, NO, and H_2_S have a complex relationship in the development of CKD, including hypertension and diabetes [255]. Decreased H_2_S has been shown to be associated with a reduction in NO production but enhanced CO production, while CO serves as a mediator between NO and H_2_S [256]. Studies have demonstrated that NO synthesis inhibition upregulated the urinary concentration and excretion rate of CO and the HO-dependent generation of CO by renal tissue in the non-diabetic rat [257], whereas diabetes increases oxidative stress and induces HO-1 protein expression (and probably by the generation of CO), which contributes to regulating renal hemodynamics in conditions of low NO bioavailability [258]. These findings imply that endogenous NO is an inhibitory regulator of renal CO generation or vice versa. It has also been demonstrated that reduced endogenous H_2_S levels impair PI3K/Akt/eNOS signaling cascades, causing hyperglycemia-induced vascular injuries [259].

It has now emerged that a detailed mechanistic insight into the biology of the gasotransmitters and renal physiology is vital to translate these gaseous molecules to be novel therapeutic agents in the control and management of DN. In this regard, although some of the vascular protective effects of acetylsalicylic acid and statins are attributed to the induction of HO-1, CO administration has not yet been used clinically. The antioxidant response of resveratrol is also partly attributed to the upregulation of HO-1, as evidenced by increased HO-1 expression in STZ-induced T1D in Sprague-Dawley rats [260]. Although the HO-1-inducing effects of resveratrol have not yet been observed in humans, it is readily available as a dietary supplement.

As discussed earlier, since sulfate-reducing bacteria produce H_2_S in the gut and significantly lower levels of H_2_S were observed in germ-free mice, the dietary supplementation of sulfate or sulfur-containing amino acids may act as natural H_2_S donors. Thus, H_2_S may be an excellent tool to treat various disease conditions, including DN, depending on the relative abundance of H_2_S availability associated with the specific disease states.

## Authors Contributions

S.K.J. and U.S. designed and wrote the manuscript draft. R.O., D.D.G. and V.R.J. contributed to the discussion, and U.S. finalized the manuscript. All authors have read and agreed to the published version of the manuscript.

## Figures and Tables

**Table 1:** Effect of gasotransmitters in diabetic nephropathy: ↑ indicates elevated gasotransmitters, ↓ indicates reduced gasotransmitters.

Gasotransmitter	Experimental Model	Intervention	Level	Outcome	Refs.
NO	STZ-induced diabetic mouse/rat	eNOS^−/−^	↓	Enhanced vascular damage and renal insufficiency	[45]
		NO donor sodium nitrite NO precursor, L-arginine	↑	Ameliorated collagen accumulation and renal function	[98]
	Lepr^db/db^ mouse	eNOS^−/−^	↓	Augmented glomerular injury, proteinuria, and renal insufficiency	[44]
	OLETF rat	NOS co-factor BH4	↑	Decreased glomerular injury and proteinuria	[47]
		L-NAME	↓	Enhanced glomerular injury, proteinuria, and inflammation	[46]
	STZ-induced diabetic rat	L-NAME	↓	Induced collagen accumulation and renal dysfunction	[98]
CO	STZ-induced diabetic mouse	HO-2^−/−^	↓	Increased renal injury and loss of renal function	[23]
		HO inducer CoPP	↑	Mitigated glomerular injury and renal insufficiency	[23]
	STZ-induced diabetic rat	HO inducers hemin and CoPP	↑	Ameliorated renal injury, inflammation, and renal function	[20,21,22]
		HO inhibitors SnMP and CrMP	↓	Increased renal injury and prevented protective effects of hemin	[20,21]
	ZDF rat	Hemin	↑	Ameliorated renal injury, inflammation, and renal function	[19]
		HO inhibitor SnMP	↓	Increased renal injury and renal insufficiency	[19]
H_2_S	C57BL/6J-Ins2^Akita^	H_2_S donor N-acetyl-cysteine	↑	Decreased ROS	[86]
		H_2_S donor GYY4137	↑	Ameliorated renal fibrosis and vasoconstriction	[99]
		H_2_S donor NaHS	↑	Mitigated renovascular remodeling and dysfunction	[1]
	STZ-induced diabetic rat	H_2_S donor NaHS	↑	Reduced ROS and autophagy and ameliorated renal injury, inflammation, fibrosis, and renal function	[88,89]

Abbreviations: STZ, streptozotocin; Leprdb/db, mice homozygous for the diabetes spontaneous mutation (Leprdb); OLETF, Otsuka Long-Evans Tokushima Fatty; ZDF, Zucker diabetic fatty; CrMP, chromium mesoporphyrin; SnMP, stannous mesoporphyrin; CoPP, cobalt protoporphyrin; NaHS, sodium hydrosulfide.

## Data Availability

All data is contained within the article.

## Abbreviations

α-SMA: alpha-smooth muscle actin; ACE, angiotensin-converting enzyme; AGEs, advanced-stage glycosylation end products; ATP, adenosine triphosphate; CAT, cysteine aminotransferase; CBS, cystathionine β-synthase; CO, carbon monoxide; CORM, carbon monoxide-releasing molecule; CsA, cyclosporin A; CSE, cystathionine γ-lyase; CTGF, connective tissue growth factor; Cx, connexin; DN, diabetic nephropathy; ECM, extracellular matrix; ESRD, end-stage renal disease; GBM, glomerular basement membrane; GFR, glomerular filtration rate; H_2_S, hydrogen sulfide; HO, heme oxygenase; KO, knockout; LTP, long-term potentiation; 3-MST, 3-mercaptopyruvate sulfurtransferase; MMP, matrix metalloproteinase; MT-MMP, membrane-type matrix metalloproteinase; NaHS, sodium hydrosulfide; NMDA-R, N-methyl-D-aspartate-receptor; NO, nitric oxide; NOS, nitric oxide synthase; PDGF, platelet-derived growth factor; PLP, pyridoxal phosphate; PPARγ, peroxisome proliferator-activated receptor gamma; ROS, reactive oxygen species; SLR, small leucine-rich; SNP, sodium nitroprusside; STZ, streptozotocin; T1D/T2D, type 1/type 2 diabetes; TBM, tubular basement membrane; TGF-β, transforming growth factor-β; TIMP, tissue inhibitor of matrix metalloproteinase; TLR-4, toll-like receptor-4; TZDs, thiazolidinediones; VSMC, vascular smooth muscle cell.
